# Supplementary material for: Symptoms of fatigue and depression is reflected in altered default mode network connectivity in multiple sclerosis
Source: PLoS One. 2019 Apr 1;14(4):e0210375. doi: 10.1371/journal.pone.0210375 (PMC6443168; doi:10.1371/journal.pone.0210375)
Supplement: S1 File — (DOCX) [file pone.0210375.s001.docx]

# **Supporting Information**

Fig. 2 provides an overview of the first six PCA components, which in total explain 64 % of the total variance in the data. Some potentially interesting patterns may be noted: PCA-3 shows positive correlations with BDI items concerning appetite (item 18) and weight loss (item 19), PCA-4 shows positive correlation with a BDI item concerning suicide (item 9), PCA-5 shows positive correlation with a BDI item concerning how subjects are annoyed (item 11) and negative correlation with a BDI item concerning how often the subjects cry (item 10), PCA-6 shows positive correlation with an item concerning if the subjects are feeling like they´re being punished (item 6). In general, these findings would be interesting to investigate in a larger sample, and could potentially be of clinical interest. Given our relatively small sample, which typically renders such multivariate decompositions less robust and more vulnerable to outliers and noise, we limited our focus on the two major factors, which are likely to be more stable.

**
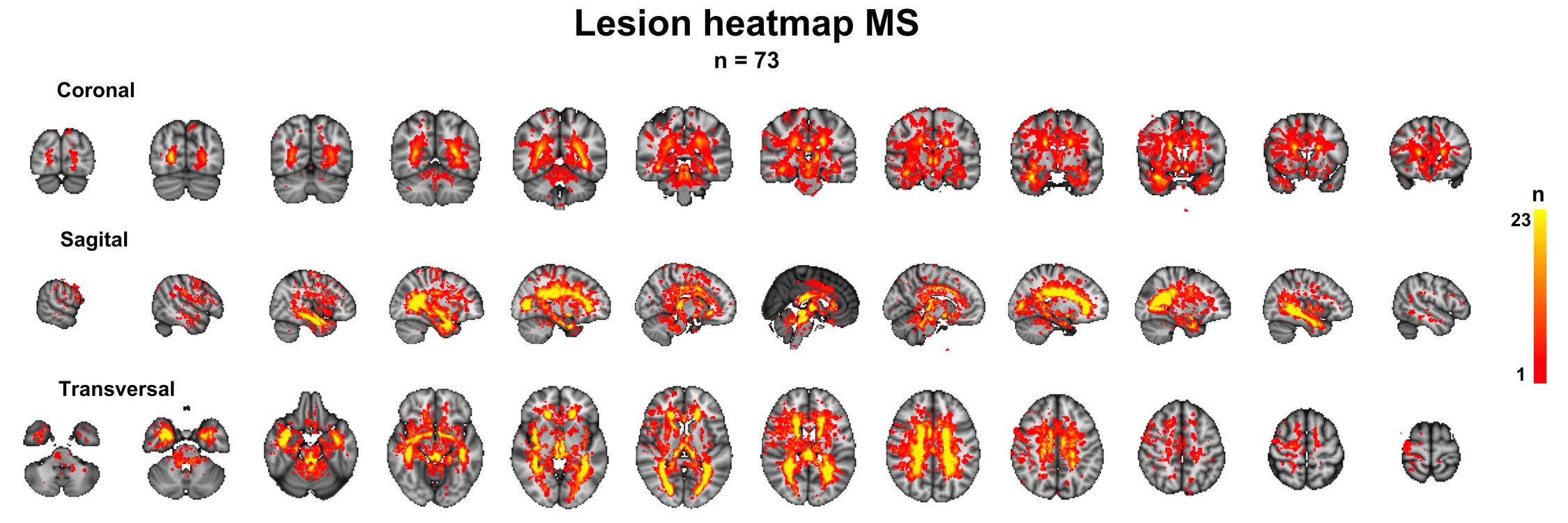
**

**S1 Fig. Heat map showing the distribution of MS lesions in the brain.** Utilizing automatically generated lesion masks from Cascade [37], we plotted all normalized MS lesions from time point 1 on the standardized MNI-152–template to visualize the distribution of the MS lesions in the brain. Depicted are slices in the coronal, sagital and transversal plane evenly distributed. Increasing yellow colour represents higher lesion count, as depicted in the colour bar.
